# Association of serum Cyr61 levels with peripheral arterial disease in subjects with type 2 diabetes

**DOI:** 10.1186/s12933-020-01171-9

**Published:** 2020-11-22

**Authors:** Bin Feng, Guidong Xu, Kangyun Sun, Kaipeng Duan, Bimin Shi, Nannan Zhang

**Affiliations:** 1 Department of Endocrinology and Metabolism, The First Affiliated Hospital of Soochow University, Jiangsu Province, Suzhou, 215006 P.R. China; 2 Department of Cardiology, The Affiliated Suzhou Hospital of Nanjing Medical University, Jiangsu Province, 242 Guangji Road, Suzhou, 215008 PR China; 3 Department of General Surgery, The First Affiliated Hospital of Soochow University, Jiangsu Province, Suzhou, 215006 P.R. China

**Keywords:** Cyr61, Type 2 diabetes, Peripheral artery disease, Atherosclerosis

## Abstract

**Background:**

The prevalence of peripheral artery disease (PAD) is obviously increased in patients with diabetes. Existing evidence shows that cysteine-rich angiogenic inducer 61 (Cyr61), a 40-kD secreted protein, plays important roles in regulating cellular physiological processes. Recent studies have demonstrated a significant correlation between serum Cyr61 and atherosclerosis. However, the relationship between Cyr61 levels and PAD in patients with type 2 diabetes (T2DM) remains obscure.

**Methods:**

Data from a total of 306 subjects with T2DM were cross-sectionally analysed. The extent of PAD was determined by using the Fontaine classification, which defines four stages. We measured serum Cyr61 concentrations by ELISA in subjects with and without PAD at Fontaine’s stage II, III, or IV. Logistic regression models were used to examine the independent association of Cyr61 with PAD.

**Results:**

Out of the 306 subjects enrolled, 150 were free from PAD, while 156 had clinically significant PAD. In subjects with PAD, the prevalences of Fontaine classification stages II, III and IV were 48.7%, 32.1%, and 19.2%, respectively. Patients with more advanced PAD had significantly higher Cyr61 (*P* for trend < 0.001). The prevalence of PAD on the basis of severity increased with increasing Cyr61 quartiles (all *P* values for trends < 0.001), and the severity of PAD was positively correlated with Cyr61 quartiles (r = 0.227, *P* = 0.006). The association of Cyr61 levels with PAD remained after adjusting for major risk factors in a logistic regression analysis.

**Conclusions:**

Our results demonstrated that Cyr61 was significantly increased in PAD patients with T2DM and that Cyr61 levels were positively associated with disease severity. Cyr61 could be a promising biomarker and further studies are needed to assess its clinical utility.

## Background

Peripheral artery disease (PAD) is a common manifestation of atherosclerotic disease, which is related to considerable disability and mortality. Currently, approximately 202 million people worldwide are suffering from lower extremity arterial disease, giving rise to a major public health problem and a heavy economic burden [1, 2]. Type 2 diabetes (T2DM) is one of the major risk factors for atherosclerosis, and the prevalence of PAD increases with the prevalence of T2DM. Moreover, compared with nondiabetic individuals, patients with diabetes have a poorer prognosis for PAD [1, 2]. Therefore, early diagnosis and intervention of PAD in patients with diabetes are essential to reduce the risk of major adverse limb events (MALEs) [3]. At present, various international guidelines recommend the ankle brachial index (ABI) as the preferred screening tool for PAD in diabetic individuals [4]. Due to the low sensitivity of the ABI for the detection of early-stage PAD, there is an urgent need to find novel biomarkers that can identify PAD among patients with diabetes in the initial stage.

Previous studies have found that some members of the cellular communication network (CCN) family are highly expressed in atherosclerotic plaques, contributing to the development of cardiovascular and cerebrovascular diseases and peripheral arterial diseases [5, 6]. Cysteine-rich angiogenic inducer 61 (Cyr61), belonging to the CCN family, is a 40 kD secreted extracellular matrix (ECM)- related signalling protein that can regulate cell proliferation, adhesion, differentiation and extracellular matrix production [7–9]. Cyr61 is maintained at a low level under normal conditions, but is usually elevated in various states of disease, such as colitis, rheumatoid arthritis, Graves’ orbitopathy, diabetic retinopathy and atherosclerosis [10–15]. Downregulation of Cyr61 expression in carotid balloon injury rats can mitigate the proliferation of vascular smooth muscle cells, thereby attenuating vascular intimal hyperplasia [16]. Moreover, inhibition of the Cyr61 signalling pathway contributes to the reduction in vascular smooth muscle cell senescence in human coronary artery smooth muscle cells [17]. Additionally, recent studies demonstrated that Cyr61 levels were independently associated with 30-day mortality in patients with acute heart failure (AHF) and coronary heart disease (CAD) [18], and could be a potential marker of myocardial ischemic injury and prognosis in patients with acute coronary syndrome (ACS) [9, 19]. To date, however, the link between circulating Cyr61 and PAD in patients with diabetes has not yet been established.

Since numerous studies have demonstrated links between Cyr61 and various aspects of atherosclerosis [11, 20] and diabetic microvascular complications [10], it is reasonable to hypothesize a correlation between Cyr61 and PAD in the diabetic condition. Therefore, we sought to investigate the potential role of Cyr61 as a marker of endothelial dysfunction and PAD in patients with T2DM.

## Methods

### Study population and design

We performed a cross-sectional study that was approved by the Ethics Committee of the First Affiliated Hospital of Soochow University and the Affiliated Suzhou Hospital of Nanjing Medical University in accordance with the principles of the Helsinki Declaration. Written informed consent was obtained from each participant. PASS 15.0 software (NCSS, LLC) was used to calculate the sample size. Two-sided intervals and 95% confidence were required. The expected standard deviation of serum Cyr61 was 13.0 pg/ml, and 1.5 pg/ml margin error is allowed. The calculated sample size is N = 289 for this study.

Individuals with T2DM were consecutively recruited from the Department of Endocrinology and Metabolism of the First Affiliated Hospital of Soochow University and the Department of Cardiology of the Affiliated Suzhou Hospital of Nanjing Medical University, Jiangsu, China, from 1 October 2018 to 31 March 2020. The diagnosis of diabetes is based on the 1999 World Health Organization (WHO) criteria. Inclusion criteria were age ≥ 18 years with the presence of T2DM. Exclusion criteria included renal failure with estimated glomerular filtration rate (eGFR) < 30 ml/min; acute infectious disease at the time of evaluation; a history of malignancy, mental disorders, autoimmune diseases, or severe heart or liver dysfunction; and history of solid or hematological neoplasia or active neoplasia. For each participant in the study, ankle-brachial index (ABI) measurement was performed, and according to the judgement of the attending physician, both lower limbs were further assessed using arterial Doppler ultrasound-enhanced ultrasonography, computed tomography (CT) angiography, or lower limb angiography. The participants were considered to have PAD if: they had a previous history of lower limb percutaneous transluminal angioplasty, with or without stenting; or they met at least one instrumental and one clinical criterion listed in Table 1. Subjects with an ABI > 0.90 and no clinical manifestations of PAD (intermittent claudication, resting pain, non-healing distal ulcer, or gangrene) were deemed to be without PAD and did not undergo further testing.

**Table 1:** Criteria for PAD definition in patients without a history of lower limb amputation, PTA or bypass surgery

Clinical Criteria	Instrumental criteria
Presence of Intermittent claudication	ABI < 0.90
Resting pain	TcPO_2_ < 30 mmHg
Non-healing distal ulcer	Ultrasound or angiography found atherosclerosis with stenosis, with a reduction at least 50% of the lumen diameter, consistent with clinical symptoms
Gangrene	Ultrasonographic finding of post-stenotic blood flow profile, consistent with symptoms

PTA, percutaneous transluminal coronary angioplasty

The extent of PAD was determined by using the Fontaine classification, which defines four stages: stage I, asymptomatic; stage II, intermittent claudication; stage III, resting pain; and stage IV, ischemic ulcers or gangrene [21].

### Anthropometric and biochemical measurements

Anthropometric measurements included height, weight and blood pressure. Body mass index (BMI) was defined as the weight in kilograms divided by their height (in meters) squared. From each subject, a 10-ml fasting peripheral blood sample was collected and centrifuged for 5 min at a rotating speed of 3,000. The serum was immediately frozen at − 80 °C. Total cholesterol (TC), triglycerides (TG), high-density lipoprotein cholesterol (HDL-C), and low-density lipoprotein cholesterol (LDL-C) were measured by applying standard enzymatic methods using a biochemical analyser (HITACHI 7450, Tokyo, Japan). Fasting glucose levels were determined by the glucose oxidase method. HbA_1c_ was assayed by using high-performance liquid chromatography with a VARIANT II Hemoglobin Testing System (Bio-Rad Laboratories, Hercules, CA). eGFR was calculated using the simplified Modification of Diet in Renal Disease (MDRD) formula. Serum Cyr61 was determined by a commercially available ELISA kit (R&D Systems, Minneapolis, MN) according to the manufacturer’s instructions. For each subject, the serum sample was measured twice, and the results were averaged. The hospital’s clinical laboratory center implements internal and external quality control procedures. The intra- and inter- assay coefficients of variation are presented in Additional file 1: Table S1.

### Statistical analysis

Demographic and clinical data of the groups were compared using the χ^2^ test and t-test. For non-normally distributed variables, a logarithmic transformation was carried out before further analysis. The trends of continuous variables across the various groups were assessed with the use of linear polynomial contrasts in ANOVA for normally distributed variables and the Jonckheere-Terpstra test for non-normally distributed data. The Cochran-Armitage trend test was used to examine trends of rates across groups. Status by severity of PAD was treated as an ordinal categorical variable (0 = non-PAD, 1 = Fontaine stage II, 2 = Fontaine stage III, and 3 = Fontaine stage IV). Binary logistic regression analysis was performed to investigate the effect of serum Cyr61 on the risk of PAD. The area under the receiver operating characteristic (ROC) curve was calculated to test the discrimination of PAD. Graphs were created using Prism 8.0 (GraphPad Software), and statistical analysis was performed with GraphPad Prism. A *P* value < 0.05 was considered to be statistically significant.

## Results

The clinical characteristics of the subjects are shown in Table 2. Among the 306 subjects with diabetes enrolled in the study, 156 were included as PAD, and 150 as no PAD. The mean ± SD age of the enrolled participants was 61.2 ± 11.4 years; they had a mean ± SD diabetes duration of 8.6 ± 5.6 years, fasting glucose of 7.4 ± 1.6 mmol/L and HbA_1c_ of 8.8 ± 1.7%. There were no significant differences between groups regarding BMI (*P* = 0.640), smoking status (*P* = 0.953), blood pressure (*P* = 0.344), TC (*P* = 0.172), LDL-C (*P* = 0.557) or TG (P = 0.692). In individuals with PAD, the prevalences of Fontaine classification stage II, III and IV were 48.7%, 32.1%, and 19.2%, respectively. Serum Cyr61 concentrations differed significantly across the various groups and increased progressively across the severity of PAD (*P* for trend < 0.001). Individuals with more severe PAD had a longer diabetes duration; higher HDL-C, fasting glucose and HbA_1c_ levels; and lower eGFR. They were more likely to be treated with insulin and had a higher propensity to receive renin–angiotensin–aldosterone system (RASS) inhibitors (Table 2).

**Table 2:** Demographic and clinical data of subjects with diabetes by the presence and severity of PAD

Variables	All subjects (n = 306)	No PAD (n = 150)	Fontaine classification			*P* value for trend
			II (n = 76)	III (n = 50)	IV (n = 30)	
Male	52.9	45.3	47.4	44.0	53.3	0.606
Age (years)	61.2 ± 11.4	60.4 ± 12.0	59.2 ± 11.0	61.2 ± 11.0	62.6 ± 10.8	0.233
BMI (kg/m^2^)	25.2 ± 3.3	25.1 ± 3.4	25.1 ± 3.4	25.0 ± 3.5	24.8 ± 3.3	0.640
Current smoker	26.1	25.3	28.9	24.0	26.7	0.953
Hypertension	51.0	48.0	52.6	56.0	53.3	0.344
Diabetes duration (years)	8.6 ± 5.6	7.3 ± 6.1	9.6 ± 6.4	10.7 ± 6.0	12.8 ± 5.8	< 0.001
Total cholesterol (mmol/L)	4.5 ± 1.3	4.6 ± 1.3	4.5 ± 1.3	4.7 ± 1.3	4.9 ± 1.1	0.172
TG (mmol/L)	1.8 ± 1.5	1.9 ± 1.7	1.8 ± 1.5	1.7 ± 1.4	1.8 ± 1.7	0.692
HDL-C (mmol/L)	1.2 ± 0.4	1.1 ± 0.4	1.2 ± 0.4	1.2 ± 0.4	1.3 ± 0.3	0.015
LDL-C (mmol/L)	3.1 ± 0.7	3.1 ± 0.9	3.1 ± 0.7	3.1 ± 0.7	3.2 ± 0.8	0.557
HbA_1c_ (%)	8.8 ± 1.7	8.2 ± 1.5	8.9 ± 1.6	8.9 ± 1.5	9.1 ± 1.7	0.006
Fasting glucose (mmol/L)	7.4 ± 1.6	7.1 ± 1.5	7.9 ± 1.7	7.7 ± 1.5	8.1 ± 1.5	0.004
eGFR (ml/min/1.73 m^2^)	79.4 ± 8.4	82.3 ± 8.7	79.5 ± 8.9	78.5 ± 8.8	76.5 ± 8.4	< 0.001
Serum Cyr61 (pg/ml)	219.1 ± 13.5	195.4 ± 11.9	221.0 ± 14.4	247.6 ± 11.8	275.5 ± 12.3	< 0.001
Use antidiabetes agents						
Oral drugs	58.2	61.3	60.5	48.0	55.3	0.225
Insulin	64.7	54.7	68.4	76.0	86.7	< 0.001
Use antihypertension agents						
β-Blockers	13.1	10.7	13.2	16.0	20.0	0.123
Calcium-channel blockers	24.2	24.0	23.7	24.0	26.7	0.826
RAAS inhibitors	47.1	40.0	47.4	56.0	66.7	0.003
Diuretics	6.5	5.3	7.9	8.0	6.7	0.551
Use lipid-lowering agents						
Statins	24.8	3221.3	2026.3	1428.0	1033.3	0.119
Fibrates	7.2	85.3	810.5	48.0	26.7	0.530

Data are the mean ± SD or percentage unless otherwise indicated

*BMI* body mass index, * RAAS* renin–angiotensin–aldosterone system

**Fig. 1:**
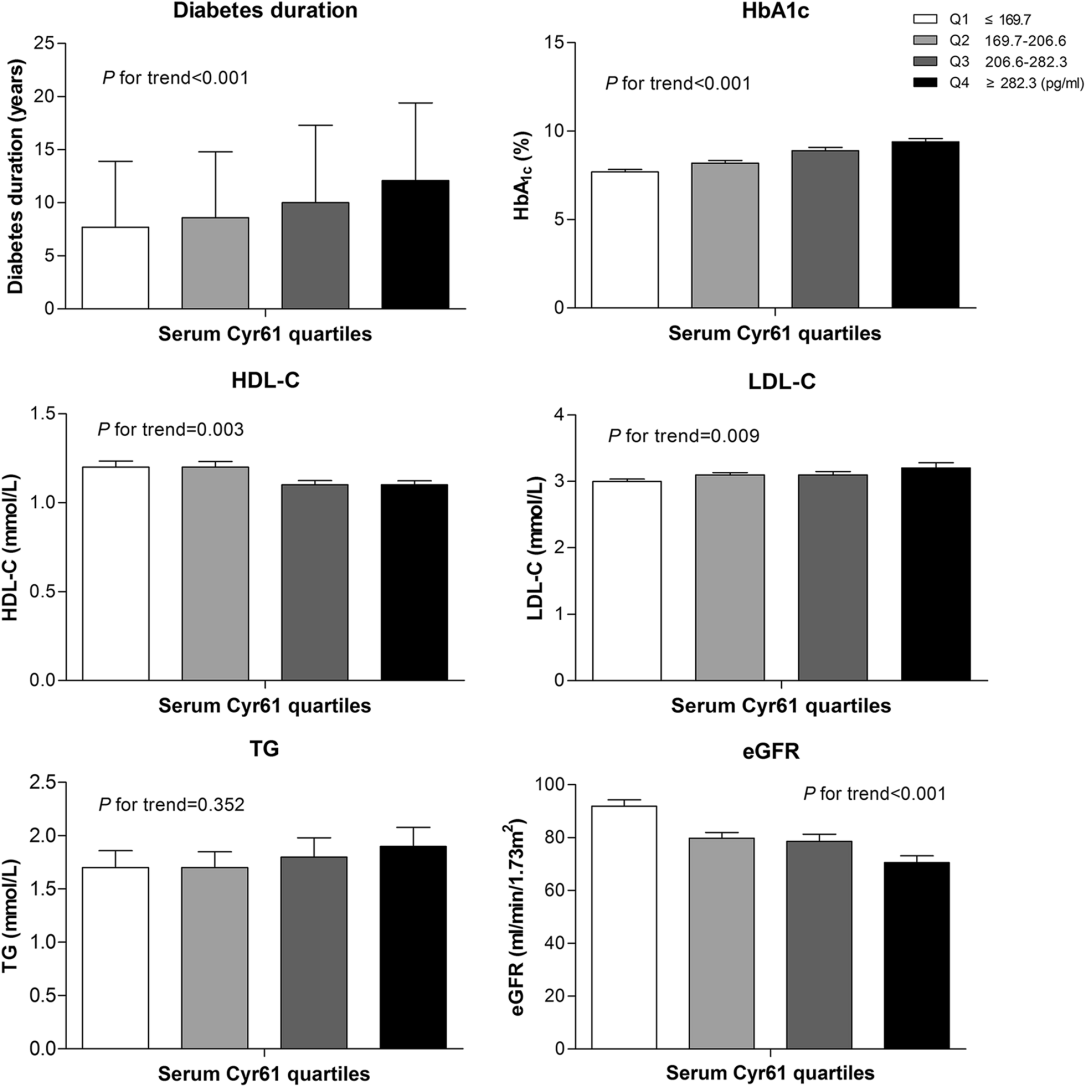
The main characteristics of study participants by quartiles of serum Cyr61. Data are the mean ± SD

**Fig. 2:**
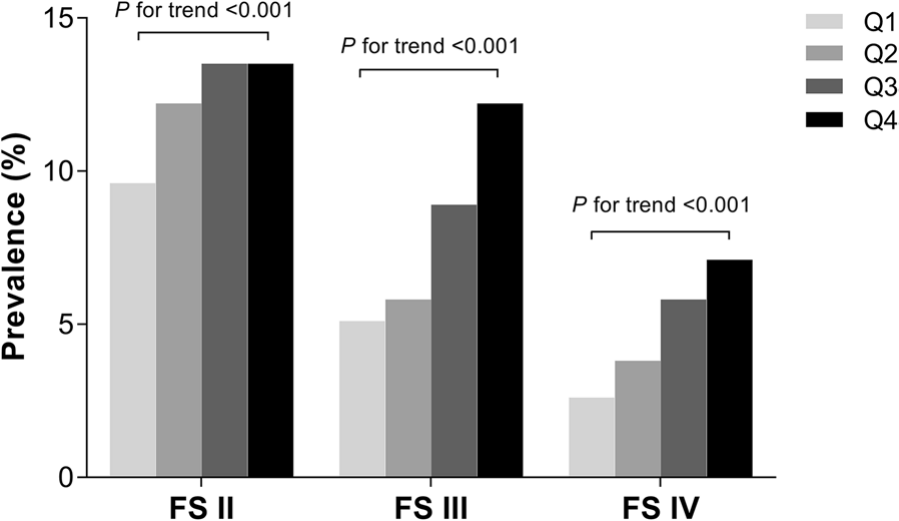
Prevalence of PAD by severity as a function of Cyr61 quartile. Cyr61 quartile: Q1: ≤ 169.7 pg/ml, Q2: 169.7–206.6 pg/ml, Q3: 206.6–282.3 pg/ml, Q4: ≥ 282.3 pg/ml. *FS* Fontaine Stage

Next, all of the participants were stratified according to quartiles of serum Cyr61 level (quartile 1 [Q1]: ≤ 169.7 pg/ml; quartile 2 [Q2]: 169.7–206.6 pg/ml; quartile 3 [Q3]: 206.6–282.3 pg/ml; and quartile 4 [Q4]: ≥ 282.3 pg/ml). As shown in Fig. 1, individuals with a longer diabetes duration have a higher serum Cyr61 concentration. Levels of HbA_1c_ and LDL-C were increased, meanwhile, levels of HDL-C and eGFR were significantly decreased in subjects of the higher Cyr61 quartile. In general, the prevalence of PAD by severity increased with ascending quartiles of Cyr61 (all *P* values for trends < 0.001) (Fig. 2). A significant association between the Cyr61 quartile and the severity of PAD was observed after Spearman correlation analysis (r = 0.227; *P* = 0.006). More detailed characteristics are presented in Additional file 2: Table S2.

Logistic regression analysis revealed that serum Cyr61 was significantly associated with the risk of PAD in both the crude and adjusted models including age, sex, BMI, diabetes duration, fasting glucose, hypertension, TG, HDL, LDL, HbA1c, eGFR, smoking status, use of RAAS inhibitors, statins and fibrates as covariates (all *P* values < 0.005, Table 3).

**Table 3:** Association of serum Cyr61 with PAD by logistic regression analyses.

Modes	OR	95% confidence interval	*P* value
Crude	0.915	0.854—0.949	< 0.001
Mode1	0.923	0.867—0.964	0.005
Mode2	0.936	0.872—0.981	0.008
Mode3	0.947	0.879—0.993	0.048

Model 1 is adjusted for age, sex and BMI; Model 2 includes all variables in Model 1 plus diabetes duration, fasting glucose, hypertension, TG, HDL-C and LDL-C; Model 3 includes all variables in Model 2 plus HbA1c, eGFR, smoking status, use of RAAS inhibitors, statins and fibrates

**Fig. 3:**
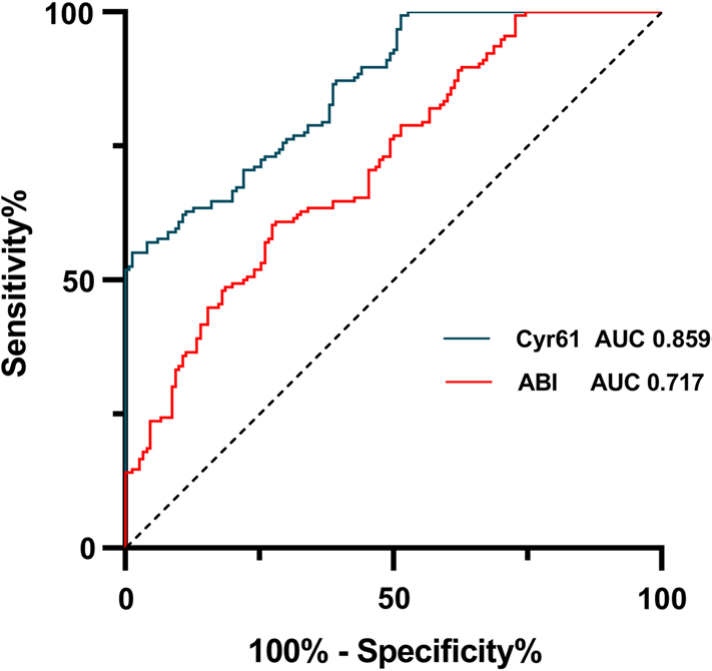
Receiver operating characteristic (ROC) curves for Cyr61 and ABI in 306 subjects with diabetes. The curves show discriminative capability for determination of PAD between Cyr61 and ABI measurements. *ROC* receiver operating characteristic, *AUC* area under the curve

Receiver-operator characteristic (ROC) curves were generated to evaluate the discriminative capability of Cyr61 for determination of PAD and to compare it with the ABI. In all subjects with diabetes, the area under the curve (AUC) for Cyr61 was significantly greater than that for the ABI (Fig. 3). The best cut-off value of Cyr61 for PAD detection in the present study was > 233.7 ng/ml (sensitivity 82.7%, specificity 72.3%).

## Discussion

Peripheral arterial disease usually seriously affects the daily quality of life of patients and imposes huge personal and societal healthcare burdens [22, 23]. To the best of our knowledge, this is the first study to evaluate the association between serum Cyr61 levels and the risk of PAD. However, due to the cross-sectional nature of the study design, it is impossible to determine the causality between Cyr61 and PAD. Thus, we have to be careful about interpreting the associations concluded from this survey.

### Potential biomarkers for diabetes with PAD

Considering the relevance of the disease, effective earlier screening and diagnosis of PAD for patients with diabetes has become increasingly important. However, there is still a lack of effective biomarkers with high sensitivity and specificity for early diagnosis, and often a patient consults the specialist when the disease is already significantly worsened. Several potential biomarkers were found to identify PAD in patients with diabetes. For instance, serum high mobility group box (HMGB) 1, fibroblast growth factor (FGF) 23 and osteopontin (OPN) have been correlated with the presence of PAD [24, 25]. In addition, thrombospondin-4 (TSP-4) levels were significantly increased with PAD severity in patients with concomitant diabetes and could be a novel marker of atherosclerotic activity [26]. Recently, the association between circulating serum growth differentiation factor (GDF) 15 and lower extremity atherosclerotic disease (LEAD) has been investigated in Chinese diabetes subjects [27]. Furthermore, Hayashi et al. reported a method for accurate skin temperature measurement using noncontact, handheld infrared skin thermometer, which could serve as a new, cost-effective screening strategy for earlier diagnosis of PAD [28]. Very recently, Biscetti and colleagues reported that elevated plasma sortilin is significantly and independently associated with the presence of lower limb PAD in statin-free patients with diabetes, while omentin-1 is reduced in the same population. Both of them could be promising markers for clinically significant atherosclerosis in the lower limbs [29, 30].

### Cyr61 and peripheral vascular injuries

Cyr61 is a multifunctional matricellular protein that plays essential roles in regulating inflammation, wound healing and fibrogenesis in adults. Aberrant Cyr61 expression is associated with numerous pathologies, including diseases associated with autoimmune, metabolism and chronic inflammation [31]. Furthermore, regulation of the Cyr61 level in the circulation and vital organs has also been observed during experimentally induced sepsis [32, 33]. In terms of peripheral vascular disease, previous studies have demonstrated that Cyr61 is overexpressed in vascular smooth muscle cells of atherosclerotic lesions both in humans and in animal models [20, 34, 35] and could stimulate adhesion of vascular smooth muscle cells in a dose-dependent manner [11]. Interestingly, plasminogen (Plg)-mediated Cyr61 cleavage and activation promoted endothelial cell migration and neovascularization in vitro and in vivo. Targeting Plg/Cyr61 may offer exciting therapeutic opportunities for strengthening mesenchymal stem cells therapy in ischemic diseases [36]. Because the concept that inflammation participates pivotally in the pathogenesis of atherosclerosis has gained considerable attention [37], we speculated that chronic low-grade inflammation, together with the increased Cyr61 concentration contribute, at least in part, to the occurrence of atherosclerotic lesions of the lower limbs in patients with diabetes. In the present study, we found that serum Cyr61 levels increase according to severity of PAD, as if there were a dose-dependent relationship. Furthermore, results of ROC curve analysis show that Cyr61 concentrations can effectively identify the presence of PAD in subjects with diabetes, and the diagnostic sensitivity and specificity are better than the ABI. Thus, serum Cyr61 may be a promising biomarker for early diagnosis and effective follow-up of PAD in patients with diabetes.

It is not possible to definitively clarify whether Cyr61 levels are a cause or an effect of PAD, or even both, through a vicious cycle. In fact, earlier studies on Cyr61 highlighted its causative roles in intima proliferation. For instance, negative regulation of the Cyr61 gene led to the inhibition of vascular smooth muscle cell proliferation and neointimal hyperplasia [11]. Matsumae and colleagues also demonstrated that knockdown of Cyr61 significantly suppressed neointimal hyperplasia in a rat carotid artery balloon injury model [16]. Thus, targeting circulating Cyr61 may provide a novel strategy for the prevention of peripheral arterial disease.

## Limitations

The study has several major limitations that should be noted. First, due to the cross sectional design of the study, we could not establish a causal relationship between serum Cyr61 and the development of PAD. Nevertheless, our results support the hypothesis that Cyr61 is important in peripheral atherosclerosis physiopathology. Second, the cross-sectional measurements may not represent the participant’s stable level of serum Cyr61. Therefore, the results of this study should be interpreted with caution. Third, the enrolled individuals were hospitalized patients with diabetes in the Suzhou area (Jiangsu, China), and the generalizability of the results to other diabetic populations needs to be tested. A further limitation is that we need prospective data to confirm whether higher Cyr61 levels may suffice as an effective biomarker for PAD in patients with diabetes. Last, because the enrolment of participants was restricted to patients with T2DM with higher susceptibility for developing PAD, selection bias of the study was an inevitable and non-negligible issue. In addition, we did not use healthy controls to determine the normal level of Cyr61. In fact, there is still a lack of studies which focused on differences of Cyr61 levels among health populations due to aging, ethnic variation, genetic background, and nutritional status. We believe that our results can provide a valuable reference for future large-scale studies.

## Conclusion

In summary, we provided evidence that circulating Cyr61 levels are significantly correlated with the disease severity of PAD in the diabetes population. Although our findings underscore the value of Cyr61 as a biomarker, more prospective studies and clinical trials are warranted to better elucidate the role of Cyr61 in the onset and progression of PAD in T2DM patients. In addition, basic research is urgently needed to explore the underlying mechanisms of Cyr61 regulation under PAD conditions.

## Supplementary information

**Additional file 1: Table S1.** The assay coefficients of variation for measuring biochemical parameters.

**Additional file 2: Table S2.** Characteristics of study participants by quartiles of serum Cyr61. Data are the mean ± SD or percentage unless otherwise indicated. *BMI* body mass index; *RAAS* renin-angiotensin-aldosterone system.

## Data Availability

The datasets used and/or analysed for this study are available from the corresponding author upon reasonable request.

## Abbreviations

PAD: Peripheral artery disease
T2DM: Type 2 diabetes
MALEs: Major adverse limb events
CCN: Cellular communication network
AHF: Acute heart failure
CAD: Coronary heart disease
ACS: Acute coronary syndrome
eGFR: Estimated glomerular filtration rate
ABI: Ankle-brachial index
CT: Computed tomography
BMI: Body mass index
TC: Total cholesterol
TG: Triglyceride
HDL-C: High-density lipoprotein cholesterol
LDL-C: Low-density lipoprotein cholesterol
RAAS: Renin–angiotensin–aldosterone system
ROC: Receiver operating characteristic
AUC: Area under the curve
HMGB: High mobility group box
FGF: Fibroblast growth factor
OPN: Osteopontin
Plg: Plasminogen
TSP-4: Thrombospondin-4
GDF: Growth differentiation factor
LEAD: Lower extremity atherosclerotic disease
