# Supplementary material for: Association of serum Cyr61 levels with peripheral arterial disease in subjects with type 2 diabetes
Source: Cardiovasc Diabetol. 2020 Nov 22;19:194. doi: 10.1186/s12933-020-01171-9 (PMC7680586; doi:10.1186/s12933-020-01171-9)
Supplement: Supplementary file 1 — Additional file 1: Table S1. The assay coefficients of variation for measuring biochemical parameters. [file 12933_2020_1171_MOESM1_ESM.docx]

| Parameters | Laboratory reference ranges | Coefficient of variation (%) | |
| --- | --- | --- | --- |
|  |  | Intra assay | Inter assay |
| TC (mmol/L) | <5.2 | 2.5 | 3.7 |
| TG (mmol/L) | <1.7 | 4.0 | 3.3 |
| HDL-C (mmol/L) | ≥1.0 | 2.8 | 4.2 |
| LDL-C (mmol/L) | <3.4 | 3.8 | 3.3 |
| HbA_1c_ (%) | 5.1-6.0 | 3.6 | 4.0 |
| Fasting glucose (mmol/L) | 3.9-6.1 | 2.9 | 2.1 |
| Serum Cyr61 (pg/ml) | 33.0-410.0 | 6.4 | 7.6 |

**Table S1. The assay coefficients of variation for measuring biochemical parameters**
