# Supplementary material for: Association of serum Cyr61 levels with peripheral arterial disease in subjects with type 2 diabetes
Source: Cardiovasc Diabetol. 2020 Nov 22;19:194. doi: 10.1186/s12933-020-01171-9 (PMC7680586; doi:10.1186/s12933-020-01171-9)
Supplement: Supplementary file 2 — Additional file 2: Table S2. Characteristics of study participants by quartiles of serum Cyr61. Data are the mean ± SD or percentage unless otherwise indicated. BMI body mass index; RAAS renin-angiotensin-aldosterone system. [file 12933_2020_1171_MOESM2_ESM.docx]

| Variables | Serum Cyr61 quartiles | | | | | | | |  |
| --- | --- | --- | --- | --- | --- | --- | --- | --- | --- |
|  | Q1  (n=77) | Q2  (n=89) | | | Q3  (n=69) | | Q4  (n=71) | | *P* value  for trend |
| Serum Cyr61 (pg/ml) | ≤169.7 | 169.7-206.6 | | | 206.6-282.3 | | ≥282.3 | |  |
| Male | 49.4 | 46.1 | | | 44.9 | | 45.1 | | 0.589 |
| Age (years) | 61.4±12.0 | 62.6±11.1 | | | 61.2±11.1 | | 59.6±11.3 | | 0.251 |
| BMI (kg/m^2^) | 25.0±3.0 | 25.0±3.3 | | | 25.3±3.3 | | 25.8±3.2 | | 0.106 |
| Current smoker | 23.4 | 25.8 | | | 27.5 | | 28.2 | | 0.481 |
| Hypertension | 51.9 | 49.4 | | | 52.2 | | 50.7 | | 0.974 |
| Diabetes duration (years) | 7.7±6.2 | 8.6±6.2 | | | 10.0±7.3 | | 12.1±7.3 | | <0.001 |
| Total cholesterol (mmol/L) | 4.6±1.1 | 4.6±1.1 | | | 4.7±1.0 | | 4.9±1.2 | | 0.117 |
| TG (mmol/L) | 1.7±1.4 | 1.7±1.4 | | | 1.8±1.5 | | 1.9±1.5 | | 0.352 |
| HDL-C (mmol/L) | 1.2±0.3 | 1.2±0.3 | | | 1.1±0.2 | | 1.1±0.2 | | 0.003 |
| LDL-C (mmol/L) | 3.0±0.3 | 3.1±0.3 | | | 3.1±0.4 | | 3.2±0.7 | | 0.009 |
| HbA_1c_ | 7.7±1.1 | 8.2±1.3 | | | 8.9±1.5 | | 9.4±1.5 | | <0.001 |
| Fasting glucose (mmol/L) | 7.2±1.2 | 7.7±1.5 | | | 7.8±1.6 | | 8.0±1.5 | | 0.001 |
| eGFR (ml/min/1.73 m^2^) | 81.9±8.4 | 79.8±8.6 | | | 78.6±8.4 | | 76.5±8.2 | | <0.001 |
| Use antidiabetes agents |  | |  |  | |  | |  | |
| Oral drugs | 62.3 | 58.4 | | | 57.9 | | 53.5 | | 0.295 |
| Insulin | 49.4 | 58.4 | | | 72.4 | | 81.7 | | <0.001 |
| Use antihypertension agents |  | |  |  | |  | |  | |
| β-Blockers | 11.7 | 13.5 | | | 14.5 | | 12.7 | | 0.819 |
| Calcium-channel blockers | 23.4 | 23.6 | | | 23.2 | | 26.8 | | 0.663 |
| RAAS inhibitors | 37.7 | 43.8 | | | 52.2 | | 56.3 | | 0.013 |
| Diuretics | 3.9 | 5.6 | | | 7.2 | | 5.6 | | 0.563 |
| Use lipid-lowering agents |  | |  |  | |  | |  | |
| Statins | 22.1 | 25.8 | | | 26.1 | | 25.4 | | 0.655 |
| Fibrates | 3.9 | 8.9 | | | 7.2 | | 8.5 | | 0.380 |

**Table S2. Characteristics of study participants by quartiles of serum Cyr61.** Data are the mean ± SD or percentage unless otherwise indicated. BMI, body mass index; RAAS, renin-angiotensin-aldosterone system.
